# Engineering *E. coli* for simultaneous glucose–xylose utilization during methyl ketone production

**DOI:** 10.1186/s12934-018-0862-6

**Published:** 2018-01-27

**Authors:** Xi Wang, Ee-Been Goh, Harry R. Beller

**Affiliations:** 1 Joint BioEnergy Institute (JBEI), 5885 Hollis St., Emeryville, CA 94608 USA; 2 Biological Systems & Engineering Division, Lawrence Berkeley National Laboratory, Berkeley, CA 94720 USA; 3 Earth & Environmental Sciences, Lawrence Berkeley National Laboratory, Berkeley, CA 94720 USA

**Keywords:** Carbon catabolite repression, Methyl ketones, NADPH, cAMP receptor protein, Metabolic engineering

## Abstract

**Background:**

We previously developed an *E. coli* strain that overproduces medium-chain methyl ketones for potential use as diesel fuel blending agents or as flavors and fragrances. To date, the strain’s performance has been optimized during growth with glucose. However, lignocellulosic biomass hydrolysates also contain a substantial portion of hemicellulose-derived xylose, which is typically the second most abundant sugar after glucose. Commercialization of the methyl ketone-producing technology would benefit from the increased efficiency resulting from simultaneous, rather than the native sequential (diauxic), utilization of glucose and xylose.

**Results:**

In this study, genetic manipulations were performed to alleviate carbon catabolite repression in our most efficient methyl ketone-producing strain. A strain engineered for constitutive expression of *xylF* and *xylA* (involved in xylose transport and metabolism) showed synchronized glucose and xylose consumption rates. However, this newly acquired capability came at the expense of methyl ketone titer, which decreased fivefold. Further efforts were made to improve methyl ketone production in this strain, and we found that two strategies were effective at enhancing methyl ketone titer: (1) chromosomal deletion of *pgi* (glucose-6-phosphate isomerase) to increase intracellular NADPH supply and (2) downregulation of CRP (cAMP receptor protein) expression by replacement of the native RBS with an RBS chosen based upon mutant library screening results. Combining these strategies resulted in the most favorable overall phenotypes for simultaneous glucose–xylose consumption without compromising methyl ketone titer at both 1 and 2% total sugar concentrations in shake flasks.

**Conclusions:**

This work demonstrated a strategy for engineering simultaneous utilization of C_6_ and C_5_ sugars in *E. coli* without sacrificing production of fatty acid-derived compounds.

**Electronic supplementary material:**

The online version of this article (10.1186/s12934-018-0862-6) contains supplementary material, which is available to authorized users.

## Background

Medium-chain (e.g., C_11_–C_15_) methyl ketones are among the fatty acid-derived compounds that have been developed recently for potential application as diesel blending agents [1–5]. A methyl ketone biosynthetic pathway in *Escherichia coli* that has attained 40% of maximum theoretical yield with glucose as the sole carbon source includes the following features: (a) overproduction of β-ketoacyl-coenzyme A (CoA) thioesters achieved by modification of the β-oxidation pathway (via overexpression of native FadB and a heterologous acyl-CoA oxidase from *Micrococcus luteus*) and (b) overexpression of a native thioesterase (FadM) that is effective at hydrolyzing β-ketoacyl-CoA thioesters to β-keto acids, which are the immediate precursors of methyl ketones [2]. Performance of the methyl ketone-producing strain for this technology has thus far been optimized during growth with glucose [2], which is typically the dominant sugar in biomass hydrolysates. However, biomass hydrolysates also contain a substantial portion of hemicellulose-derived xylose (typically, the second most abundant sugar after glucose). Commercialization of the methyl ketone-producing technology would benefit from the increased efficiency resulting from simultaneous utilization of glucose and xylose [6].

A challenge in cultivating *E. coli* in growth medium containing both glucose and xylose is diauxic (phased, non-simultaneous) growth, whereby glucose must be depleted before other sugars, such as xylose, can be metabolized [7]. The underlying mechanism for diauxic growth is carbon catabolite repression (CCR), which is primarily mediated by components of the phosphoenolpyruvate (PEP): carbohydrate phosphotransferase (PTS) system. The glucose-specific EII complex of the PTS system consists of the permease, EIIBC^Glc^ (encoded by *ptsG*), and EIIA^Glc^ (encoded by *crr*), which has a primary role in modulating carbohydrate metabolism in *E. coli*. During glucose transport, EIIA^Glc^ is dephosphorylated, which prevents either the import of non-glucose sugars or their subsequent metabolism, and as a consequence, bacterial cells are devoid of the inducer for the corresponding operons; this is known as inducer exclusion [8]. One consequence of dephosphorylated EIIA^Glc^ is a decrease in levels of cyclic AMP (cAMP), which is produced from ATP by adenylate cyclase (activated by phosphorylated EIIA^Glc^). Lower levels of cAMP in turn limit the availability of cAMP–CRP, the complex between cAMP and CRP (cAMP receptor protein). The expression of genes that are involved in the catabolism of sugars other than glucose generally requires the cAMP–CRP complex and, consequently, is repressed in the presence of glucose. In addition, the arabinose transcriptional regulator (AraC) suppresses the xylose-catabolism genes *xylAB* and *xylFGH* by inhibiting the xylose transcriptional activator (XylR), which constitutes the second layer of CCR [9].

Multiple strategies have been proposed for engineering simultaneous hexose–pentose metabolism in *E. coli* by mitigating CCR, such as inactivation of *ptsG*, mutation of regulatory genes, and constitutive expression of key genes in pentose metabolism [10–16]. However, few studies have applied such strategies for mitigating CCR to production of fatty acid-based biofuels [17]. Challenges can be anticipated in combining metabolic strategies for simultaneous glucose–xylose utilization and methyl ketone overproduction, as changes in central carbon metabolism can substantially alter redox balance, which is needed for efficient conversion of carbon to targeted products [18].

In this study, we investigated the effects of engineering CCR mitigation into our best methyl ketone-overproducing *E. coli* strain (EGS1895 [2]). We chose to follow the CCR mitigation strategy recently described by Kim et al. [12], which was reported to offer advantages over other approaches, most notably, the engineered strains grow well on glucose, unlike some CCR-insensitive mutants defective in the glucose PTS system. Several rounds of engineering were required to optimize both (1) simultaneous glucose–xylose co-utilization and (2) methyl ketone production, as strategies targeting one of these outcomes often adversely affected the other. Ultimately, our results suggest the feasibility of engineering simultaneous utilization of glucose and xylose in *E. coli* along with substantial production of fatty acid-derived biofuels.

## Methods

### Strains, plasmids, and reagents

*Escherichia coli* strains and plasmids are listed in Table 1. Strains and plasmids along with their associated information (annotated GenBank-format sequence files) have been deposited in the public version of the JBEI Registry (https://public-registry.jbei.org; entries JPUB_009980 to JPUB_010000) and are physically available from the authors and/or addgene (http://www.addgene.org) upon request. Our previously developed methyl ketone-overproducing strain EGS1895 [2] was used as the control strain. Q5 High-Fidelity DNA Polymerase was used for all PCR reactions (New England Biolabs, Ipswich, MA). NEBuilder HiFi DNA Assembly Master Mix (New England Biolabs, Ipswich, MA) was used to assemble linear DNA fragments. Plasmid extractions were carried out by using QIAGEN miniprep kits (Valencia, CA). Oligonucleotide primers were synthesized by Integrated DNA Technologies, Inc. (San Diego, CA). DNA sequencing was completed by GENEWIZ (South Plainfield, NJ).

**Table 1:** Strains and plasmids used in this study

	Relevant characteristics	Source or reference
Strains		
EGS1405	*E. coli* DH1; Δ*fadE*; Δ*ackA*-*pta*; Δ*poxB*	[2]
EGS1895	EGS1405 with pEG1675	[2]
XW1003	*E. coli* DH1; Δ*fadE*; Δ*ackA*-*pta*; Δ*poxB*; Δ*ptsG*	This study
XW1004	XW1003 with pEG1675	This study
XW1013	*E. coli* DH1; Δ*fadE*; Δ*ackA*-*pta*; Δ*poxB*; P_CP6_-*xylF*; P_CP25_-*xylA*; *xylA*^*up*^	This study
XW1014	XW1013 with pEG1675	This study
XW1018	XW1013 with pXW1678	This study
XW1023	*E. coli* DH1; Δ*fadE*; Δ*ackA*-*pta*; Δ*poxB*; P_CP6_-*xylF*; P_CP25_-*xylA*; *xylA*^*up*^; Δ*araE*	This study
XW1024	XW1023 with pEG1675	This study
XW1043	*E. coli* DH1; Δ*fadE*; Δ*ackA*-*pta*; Δ*poxB*; P_CP6_-*xylF*; P_CP25_-*xylA*; *xylA*^*up*^; Δ*araE*; P_CP25_-*araB*; Δ*araC*; P_CP6_-*araF*	This study
XW1044	XW1043 with pXW1677	This study
XW1053	*E. coli* DH1; Δ*fadE*; Δ*ackA*-*pta*; Δ*poxB*; P_CP6_-*xylF*; P_CP25_-*xylA*; *xylA*^*up*^; Δ*pgi*	This study
XW1054	XW1053 with pEG1675	This study
XW1055	XW1053 with pXW1678	This study
XW1063	*E. coli* DH1; Δ*fadE*; Δ*ackA*-*pta*; Δ*poxB*; P_CP6_-*xylF*; P_CP25_-*xylA*; *xylA*^*up*^; *crp*-RBS (TIR = 13)	This study
XW1064	XW1063 with pEG1675	This study
XW1073	*E. coli* DH1; Δ*fadE*; Δ*ackA*-*pta*; Δ*poxB*; P_CP6_-*xylF*; P_CP25_-*xylA*; *xylA*^*up*^; *crp*-RBS (TIR = 13); Δ*pgi*	This study
XW1074	XW1073 with pEG1675	This study
XW1075	XW1073 with pXW1678	This study
Plasmids		
pKD13	λ-Red recombineering plasmid	[19]
pKD46	λ-Red recombineering plasmid	[19]
pCP20	λ-Red recombineering plasmid	[19]
pEG1675	Km^r^, *araC*-P_BAD_-*fadR*-*co_fadD*; P_trc_-*fadM*; P_lacUV5_-*‘tesA*-*fadB*-*co_aco*	[2]
pXW1677	pEG1675 with *araC* deleted	This study
pXW1678	Km^r^, *araC*-P_BAD_-*fadR*-*co_fadD*; P_tr_-*fadM*-*maeB*; P_lacUV5_-*‘tesA*-*fadB*-*co_aco*	This study

### Genetic manipulations and strain development

All genome engineering was conducted by using the λ-Red recombination system with vectors pKD13, pKD46, and pCP20 [19, 20]. All primers used in this study are listed in Additional file 1: Table S1. CCR mitigation strategies developed by Kim et al. [12] (Table 2) were used to engineer the simultaneous utilization of glucose and xylose in strain EGS1895. Specifically, the synthetic constitutive promoters CP6 and CP25 [21] were used to replace native promoters for key genes in pentose transport (*araFGH* and *xylFGH*) and catabolism (*araBAD* and *xylAB*), respectively. The arabinose transcription factor *araC* was deleted from the chromosome. Additionally, the arabinose-proton symporter *araE* was inactivated and a point mutation was introduced in the 5′-flanking region of *xylA* (*xylA*^*up*^). We also deleted *araC* from the methyl ketone-pathway plasmid pEG1675 [2]. By inserting an *araC*-free fragment between *Spe*I and *Age*I restriction sites of pEG1675, a new plasmid, pXW1677, was created. The resultant strain with pXW1677 was named XW1044 (Tables 1, 2), and the two intermediate strains were named XW1014 and XW1024 (Tables 1, 2). For constructing the pXW1678 plasmid, the *E. coli* DH1 native *maeB* gene was cloned and inserted downstream of *fadM* at an SalI restriction site on pEG1675.

**Table 2:** Strategies used for engineering simultaneous glucose–xylose utilization in EGS1895

No.	Manipulation	Target sequence	XW1014	XW1024	XW1044
1	Replace promoter of *xylFGH*	CP6 synthetic promoter	+^a^	+	+
2	Replace promoter of *xylAB*	CP25 synthetic promoter	+	+	+
3	*xylA*^*up*^ mutation	G (+6) → T of CP25	+	+	+
4	*araE* inactivation	Truncated *araE*		+	+
5	Replace promoter of *araFGH*	CP6 synthetic promoter			+
6	Replace promoter of *araBAD*	CP25 synthetic promoter			+
7	Chromosomal deletion of *araC*	Δ*araC* (genome)			+
8	*araC* deletion from pEG1675	Δ*araC* (plasmid)			+

Based on [12]

^a^+ indicates that the specified genetic modification is present in this strain

Studies were conducted to modulate and optimize the expression of the CRP by testing a set of *crp* ribosomal binding sites (RBS). To mutate the RBS for CRP, the RBS Library Calculator [22] was used to design RBS mutant library sequences and λ-Red recombineering was used to integrate the library mutants into the host genome. For RBS calculations, the λ-Red-generated 81-bp scar sequence (5′-ATTCCGGGGATCCGTCGACCTGCAGTTCGAAGTTCCTATTCTCTAGAAAGTATAGGAACTTCGAAGCAGCTCCAGCCTACA-3′) was used as the pre-sequence. Based on the native 35-bp RBS sequence for *crp* (5′-CTCTGGAGAAAGCTTATAACAGAGGATAACCGCGC-3′), a degenerate RBS sequence (5′-CTHTGGTGAAAGCTTATAACTGAGGMRAACCGCGT-3′) was generated with a broad range of predicted translation initiation rates (TIR) for a total 12 variant sequences. RBS sequences and their predicted TIR values are listed in Additional file 1: Table S2. For reference, the native *crp* RBS sequence in *E. coli* DH1 was predicted to have a TIR value of 2441 au based on the RBS Calculator [23, 24].

### Media and culture conditions

Lysogeny broth (LB) was used for routine cell growth and propagation. Kanamycin was added to the growth medium at a final concentration of 50 µg mL^−1^, when required. M9-MOPS minimal medium with 1% total sugars (5 g L^−1^ glucose and 5 g L^−1^ xylose) or 2% total sugars (10 g L^−1^ glucose and 10 g L^−1^ xylose) as carbon sources was used for production experiments. The composition of M9-MOPS minimal medium followed the recipe previously described [2]. For production experiments, strains were first adapted in M9-MOPS minimal medium with 1% total sugars for 3 passages before being inoculated into production medium with glucose and xylose.

### Analysis of cell growth and sugar metabolism

Cell growth was monitored by measuring optical density at 600 nm (OD_600_). Sugars were measured with an Agilent 1100 Series HPLC system, equipped with an Agilent 1200 Series refractive index detector (RID) (Agilent Technologies, CA) and Aminex HPX-87H ion-exclusion column (300-mm length, 7.8-mm internal diameter; Bio-Rad Laboratories, Inc., Hercules, CA). The column temperature was 50 °C, and 4 mM sulfuric acid was used as the mobile phase with a flow rate of 0.6 mL min^−1^ for 24 min. The quantification of glucose and xylose was conducted by external standard calibration with authentic standards.

### Production and analysis of methyl ketones

For methyl ketone production, strains were inoculated into 50 mL M9-MOPS minimal medium with 1% total sugars, or 25 mL with 2% total sugars, in 250-mL shake flasks and cultured at 37 °C with 200-rpm agitation. The starting OD_600_ during production was ca. 0.01. Gene expression was induced by adding 0.2 mM IPTG and 1 mM arabinose after 6 h of growth. Five mL of decane (Reagent-Plus ≥ 99% purity, Sigma-Aldrich, St. Louis, MO) amended with perdeuterated tetracosane (C_24_D_50_) and 3-tetradecanone (Sigma-Aldrich, St. Louis, MO) as internal standards was also added to the cultures during induction. The decane overlay was sampled for the measurement of methyl ketones; analysis by electron ionization gas chromatography–mass spectrometry (GC–MS) was conducted as previously described [3].

### Batch fermentation of strains EGS1895 and XW1075 in a 2-L bioreactor for methyl ketone production

Batch fermentation was carried out in a 2-L bioreactor equipped with a Sartorius BIOSTAT B plus control unit for regulating dissolved oxygen (DO), pH, and temperature. Frozen glycerol stocks of M9-MOPS-adapted cells were used to seed a test tube containing 5 mL of M9-MOPS minimal medium with 1% total sugars (5 g L^−1^ glucose and 5 g L^−1^ xylose) as previously described. After 30 h of growth, the cultures were diluted 1:250 into a 250-mL flat-bottom shake flask containing 50 mL of M9-MOPS minimal medium supplemented with 1% total sugars. This culture was grown for another 30 h as before and was used to inoculate 1.25 L of medium in the bioreactor. The medium was adapted from Korz et al. [25] and was composed of M9 salts (6.8 g L^−1^ Na_2_HPO_4_, 3.0 g L^−1^ KH_2_PO_4_, 1.0 g L^−1^ NH_4_Cl, 0.5 g L^−1^ NaCl) supplemented with 0.5 g L^−1^ of MgSO_4_·7H_2_O, 0.18 g L^−1^ of NH_4_Cl, 1.0 mg L^−1^ thiamine, 10 nM of FeSO_4_·7H_2_O, 100 µM CaCl_2_·2H_2_O, micronutrients as described previously [2], 10 g L^−1^ of glucose, 10 g L^−1^ of xylose, and 50 µg mL^−1^ of kanamycin. The temperature of the bioreactor was maintained at 37 °C throughout the fermentation and the culture was maintained at pH 6.5 automatically by the addition of a 10 M potassium hydroxide solution. The initial stir rate and airflow were set at 200 rpm and 1 VVM (volume of air per volume of liquid per minute), respectively. Dissolved oxygen was maintained above 40% of saturation via cascade control of adjustment of stirrer speed (up to 1600 rpm), followed by air-flow rate (up to 2.0 VVM). Cultures were induced with 1 mM arabinose and 0.5 mM IPTG at 6 h after initiation of batch phase. In addition, 150 mL of dodecane (Sigma-Aldrich, ReagentPlus ≥ 99% purity) amended with 3 mg mL^−1^ of 3-tetradecanone (Sigma-Aldrich) as an internal standard, was added into the bioreactors.

At selected times, 10–15-mL samples were removed from the bioreactors via a syringe affixed to the sampling tube while the stirrer was still operating. Approximately 50 µL of cell cultures were filtered through a 0.2-µm syringe membrane filter directly into a vial for HPLC analysis and the rest of the cultures transferred to a 15-mL Falcon tube. After allowing the samples to sit in the 15-mL tube for 1 min, the supernatant dodecane overlay was pipetted out into a 2-mL microcentrifuge tube and centrifuged at 21,130×*g* for 10 min to obtain a better-resolved aqueous-organic interface. The dodecane overlay was transferred into a glass vial and stored at 4 °C until GC–MS analysis.

## Results and discussion

### Engineering simultaneous glucose–xylose utilization in methyl ketone-overproducing strain EGS1895

**Fig. 1:**
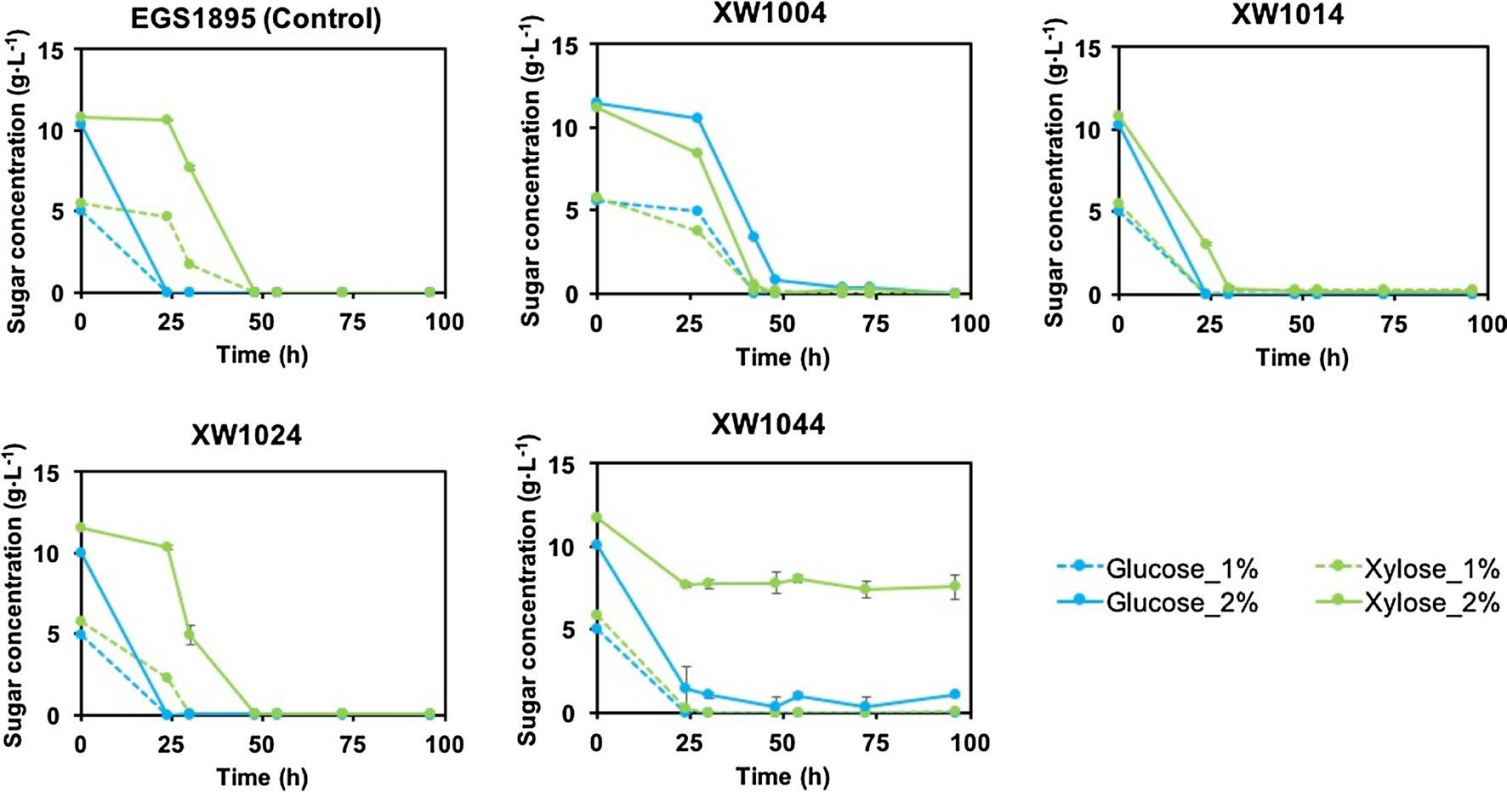
Evaluation of glucose–xylose co-utilization in engineered strains (96 h). Symbols: glucose, blue lines; xylose, green lines; 1% total sugars, dashed lines; 2% total sugars, solid lines. Error bars indicate one standard deviation (*n* = 3, except for XW1004, for which *n* = 2)

We engineered several strains by manipulating key genes in pentose metabolism (XW1014, XW1024, and XW1044; Tables 1, 2) and evaluated their ability to simultaneously utilize glucose and xylose (Fig. 1). The control strain, EGS1895, presented a typical diauxic pattern in which xylose utilization began after glucose was fully depleted. In contrast, newly engineered strains displayed glucose–xylose co-utilization to varying degrees rather than a strict diauxic profile. Among these engineered strains, XW1014 (with constitutive expression of *xylA* and *xylF* plus a point mutation in the *xylA* promoter, *xylA*^*up*^) showed the best performance for simultaneous utilization of glucose and xylose (Fig. 1). This strain had identical consumption rates for glucose and xylose at 1% sugar conditions, while a slight decrease in xylose consumption was observed at higher sugar concentration (2%). The inactivation of *araE* (XW1024; Tables 1, 2) did not result in better sugar co-utilization than was observed for strain XW1014, nor did the manipulations made for strain XW1044 (alleviating AraC-mediated repression through four collective *araC*-related manipulations, including *araC* deletion from both the genome and plasmid as well as replacement of promoters for *araB* and *araF*). Although both strains XW1024 and XW1044 showed favorable simultaneous consumption rates of glucose and xylose at 1% sugar conditions, their xylose consumption dramatically decreased at higher sugar concentration (2%).

In addition, because *ptsG* deficiency is a well-studied mechanism for mitigating CCR in *E. coli* [10], the glucose transporter EIIBC^Glc^ encoded by *ptsG* was deleted from EGS1895 to investigate the effect on sugar co-utilization (strain XW1004; Table 1). Strain XW1004 did not display a better sugar co-utilization profile than strain XW1014 (Fig. 1).

**Fig. 2:**
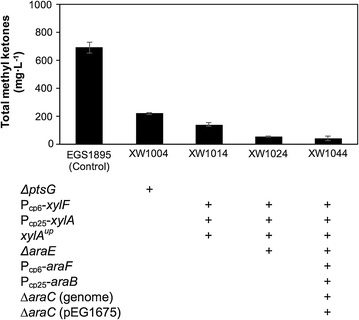
Methyl ketone production by strains engineered for glucose–xylose co-utilization (1% total sugars, 96 h). Error bars indicate one standard deviation (*n* = 3, except for XW1004, for which *n* = 2)

Methyl ketone production was also investigated among these strains engineered for hexose–pentose co-utilization. Compared with the titer of the control strain EGS1895 (~ 690 mg L^−1^), methyl ketone production was significantly reduced in all four modified strains (Fig. 2). The best performing strain for sugar co-utilization, XW1014, only produced ~ 140 mg L^−1^ total methyl ketones (1% total sugars), which is approximately fivefold lower than for strain EGS1895. Strains with more genetic manipulations produced even lower methyl ketone titers; for example, strains XW1024 and XW1044 produced < 60 mg L^−1^ methyl ketones. Although the Δ*ptsG* strain (XW1004) showed the highest methyl ketone titer among these four strains, its diminished glucose utilization was not optimal and it was not pursued further. Despite its relatively low methyl ketone titer, strain XW1014 had the most favorable combination of sugar co-utilization and methyl ketone production of the strains tested.

### Optimization of methyl ketone production in strain XW1014 by enhancing NADPH availability

Although strain XW1014 was successfully engineered for simultaneous glucose–xylose consumption, the significantly reduced methyl ketone titer in this strain necessitated further engineering to improve commercial relevance. We hypothesized that enhancing NADPH availability could be a fruitful engineering target because (1) the biosynthesis of fatty acids (methyl ketone precursors) in *E. coli* is an NADPH-demanding process and (2) xylose metabolism, particularly when simultaneous with glucose metabolism, could disrupt NADPH production in a host cell (e.g., strain XW1014) compared to conditions with glucose as a sole carbon source. Fatty acid biosynthesis results in net consumption of NADPH due to demand from two key reductases—FabG (β-ketoacyl-ACP reductase) and potentially, FabI (enoyl-ACP reductase), which can utilize either NADH or NADPH as a cofactor [1, 26]. To illustrate the substantial NADPH demands of fatty acid/methyl ketone biosynthesis, production of 1 mol of a C_13_ methyl ketone (2-tridecanone) from glucose using the relevant metabolic pathway [2] would result in net consumption of 6 (or 12) mol of NADPH and net production of 9 (or 15) mol of NADH, depending on FabI cofactor usage.

By virtue of where xylose enters central carbon metabolism in *E. coli*, xylose metabolism tends to result in less flux than glucose metabolism through the oxidative, NADPH-generating steps of the pentose phosphate pathway (PPP), namely reactions catalyzed by glucose-6-phosphate dehydrogenase (Zwf) and phosphogluconate dehydrogenase (Gnd); however, xylose metabolism can take advantage of other sources of NADPH, such as malic enzyme and transhydrogenase [27]. The situation is likely more complex when considering sugar utilization and NADPH production in strain XW1014 compared to that in control strain EGS1895. Compared with the sequential metabolism from glucose to xylose during diauxic growth (strain EGS1895), simultaneous metabolism of glucose and xylose (strain XW1014) could alter NADPH production by re-distributing flux between glycolysis and the PPP. For example, it is possible that the flux of glucose carbon through the oxidative PPP might be reduced when xylose co-utilization is occurring, because xylose metabolism will satisfy the cell’s needs for downstream PPP metabolites required for anabolism, such as erythrose 4-phosphate (needed for aromatic amino acid biosynthesis) and ribose 5-phosphate (needed for nucleic acid biosynthesis).

**Fig. 3:**
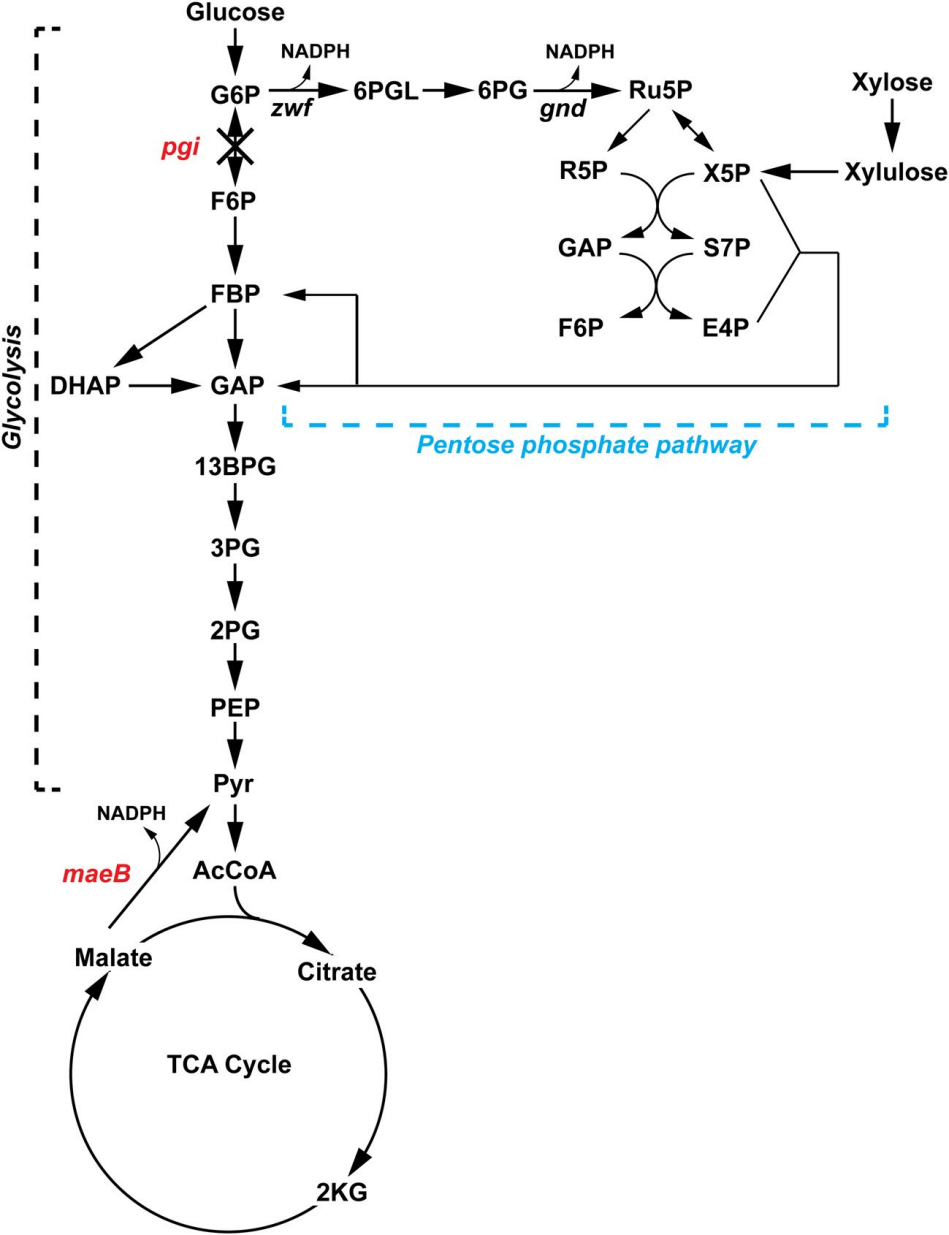
Overview of central carbon metabolism in *E. coli* highlighting strategies (in red) to improve NADPH supply in the sugar co-utilizing strain XW1014. G6P, glucose 6-phosphate; F6P, fructose 6-phosphate; FBP, fructose 1,6-bisphosphate; DHAP, dihydroxyacetone phosphate; GAP, glyceraldehyde 3-phosphate; 13BPG, 1,3-bisphosphoglycerate; 3PG, 3-phosphoglycerate; 2PG, 2-phosphoglycerate; PEP, phosphoenolpyruvate; Pyr, pyruvate; AcCoA, acetyl-CoA; 2KG: 2-ketoglutaric acid; 6PGL, 6-phosphogluconolactone; 6PG, 6-phosphogluconate; Ru5P, ribulose 5-phosphate; R5P, ribose 5-phosphate; X5P, xylulose 5-phosphate; S7P, sedoheptulose 7-phosphate; E4P, erythrose 4-phosphate; *pgi*, glucose 6-phosphate isomerase; *maeB*, malic enzyme; *zwf*, glucose 6-phosphate dehydrogenase; *gnd*, phosphogluconate dehydrogenase

We implemented two strategies for increasing NADPH supply in strain XW1014: (1) deleting *pgi* (glucose-6-phosphate isomerase) from the chromosome to divert flux from glycolysis through the oxidative PPP (Fig. 3) and (2) overexpressing *maeB* (malic enzyme), which leads to NADPH generation by oxidative decarboxylation of malate to pyruvate (Fig. 3). ^13^C Metabolic flux analysis studies in *E. coli* have shown that *pgi* deletion results in substantial production of NADPH by diversion of flux from glycolysis through the oxidative PPP, and that excessive accumulation of NADPH (cofactor imbalance) in Δ*pgi* strains can be at least partially ameliorated by NADPH consumption through transhydrogenase [28, 29]. In our Δ*pgi* strain (XW1054; Table 1), it was anticipated that a portion of the NADPH made available by the *pgi* deletion might facilitate fatty acid/methyl ketone biosynthesis by better satisfying its high NADPH demands than did central carbon metabolism in strain XW1014.

**Fig. 4:**
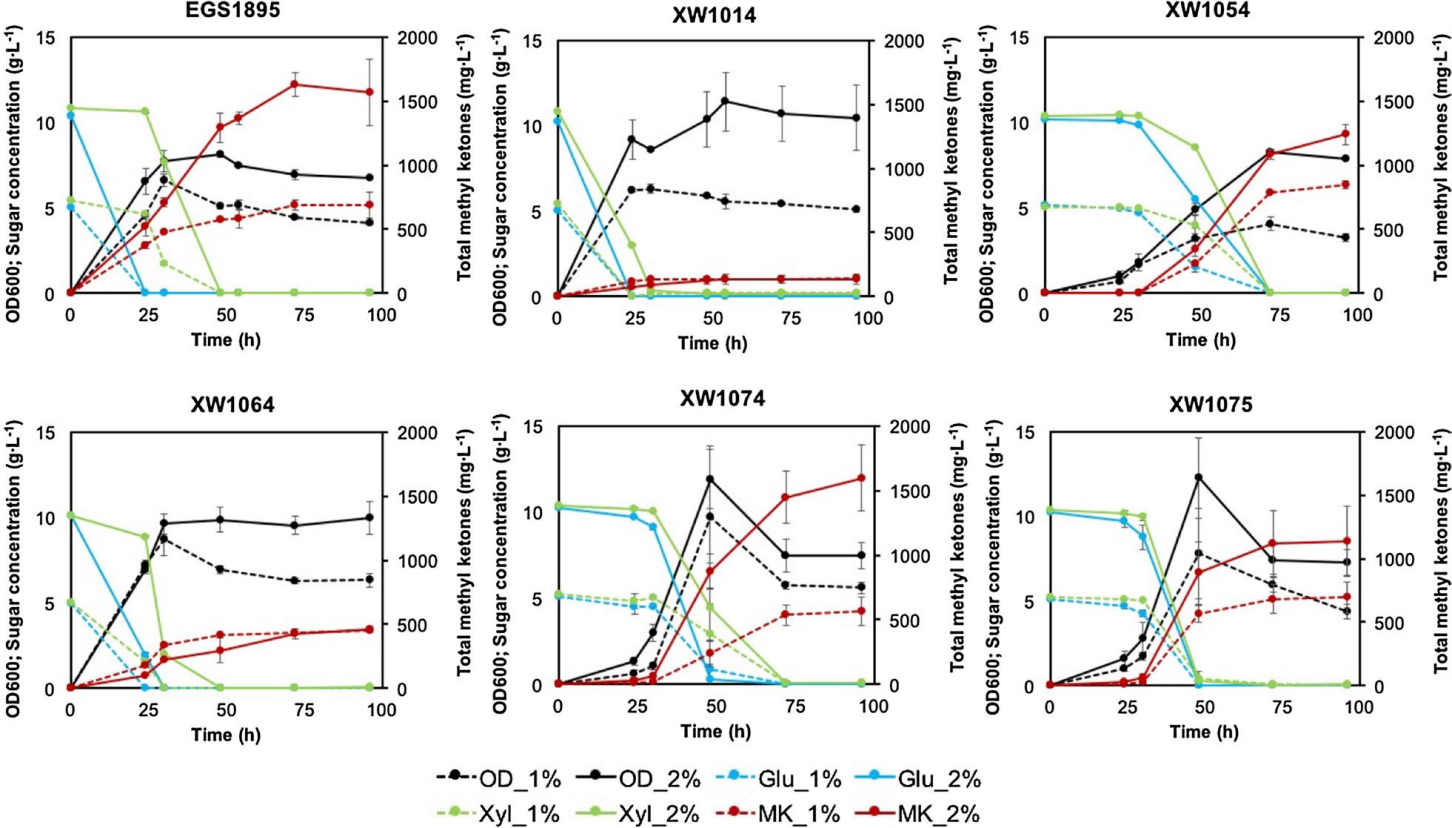
Shake flask production data (growth, methyl ketone production, sugar consumption) for strains engineered for sugar co-utilization (Table 1) and control strain EGS1895. Symbols: glucose, blue lines; xylose, green lines; OD_600_, black lines; methyl ketones, red lines; 1% total sugars, dashed lines; 2% total sugars, solid lines. The starting OD_600_ was ca. 0.01. Error bars indicate one standard deviation (*n* = 3)

Production results showed that the Δ*pgi* strain (XW1054) had dramatically improved methyl ketone titer (850 mg L^−1^) relative to strain XW1014 after 96 h at 1% total sugar conditions (Fig. 4); this methyl ketone titer was comparable to that of the control strain (EGS1895). Under 2% total sugar conditions, the methyl ketone titer of strain XW1054 (~ 1300 mg L^−1^ after 96 h) was also comparable to that of strain EGS1895 (~ 1600 mg L^−1^). However, xylose showed a slower consumption rate than glucose after *pgi* was deleted, and slower cell growth was also observed during production. In contrast to methyl ketone titer improvement for strain XW1054, the overexpression of *maeB* with or without *pgi* deletion (strains XW1055 and XW1018; Table 1) did not result in improvement in methyl ketone production (Additional file 1: Figure S1).

Based upon the results for strain XW1054 (Δ*pgi*), it is possible that NADPH is more limiting when xylose is used as a carbon source. Indeed, we observed that the control strain (EGS1895) produced very low methyl ketone titers when xylose was used as the sole carbon source in minimal medium (Additional file 1: Figure S2).

### Optimization of methyl ketone production in strain XW1014 by mutating the RBS of *crp*

While enhancing potential NADPH supply (via *pgi* deletion) substantially improved methyl ketone production with mixed glucose–xylose medium, several lines of evidence suggested that the engineered strains were experiencing suboptimal sugar utilization (e.g., strain XW1054 in Fig. 4), and potentially, suboptimal methyl ketone production, that had causes beyond NADPH limitation. For example, NADPH limitation alone does not seem to explain the dramatic reduction in methyl ketone titer in both strain XW1004 (Δ*ptsG*) and strain XW1014 (introduced constitutive promoters to *xylA* and *xylF*) (Fig. 2), as these genetic modifications are not clearly linked to NADPH supply.

A possible explanation for these results is changes in intracellular distributions of the global regulator CRP. For strain XW1014, promoter replacement for *xylA* and *xylF* resulted in removal of a CRP binding site from the intergenic region between *xylA* and *xylF* [30]. As a global regulator, CRP not only plays an important role in carbon catabolite repression, but also controls the transcription of more than 100 genes in *E. coli*, such as key genes in fatty acid metabolism (e.g., *fadD, fadH*) [31] and in central carbon metabolism (e.g., *pgi*, *zwf*, *gnd*) [30, 32]. Thus, the promoter change in strain XW1014 might have altered the level of free CRP and directly and indirectly affected the transcription of many other genes related to fatty acid metabolism. Similarly, changes to intracellular CRP pools might also explain why methyl ketone production was reduced in the Δ*ptsG* strain (XW1004): the absence of PtsG likely increased cAMP availability [33], and in turn, altered the level of free intracellular CRP, which interacts with cAMP to make the cAMP–CRP complex.

Based on this reasoning, one possible strategy for improving methyl ketone production is to optimize the expression level of CRP in strain XW1014. We attempted to modulate CRP availability by replacing the native *crp* RBS with synthetic RBSs of varying strengths. We created a mutant *crp* RBS library with broad range of predicted TIR values (8–7290 au, Additional file 1: Table S2). A total of 7 RBS variants with different TIRs were identified by sequencing from the mutant library. Screening of this library was conducted with 5-mL cultures in M9-MOPS medium (50-mL test tubes), and one mutant (strain XW1064) was selected that showed significant improvement in methyl ketone production (~ 900 mg L^−1^ after 96 h with 1% total sugars, Additional file 1: Figure S3). Notably, the predicted TIR of strain XW1064 was 13 au, which is approximately 188-fold lower than the predicted native TIR (2441 au) of *crp*. Scaled up production of strain XW1064 in 250-mL shake flasks resulted in methyl ketone titers up to ~ 450 mg L^−1^ without compromised cell growth (Fig. 4).

This result supported our hypothesis that optimized expression of CRP is able to improve methyl ketone production in the strains engineered for glucose–xylose co-utilization. However, we also noticed that the consumption rate of xylose in strain XW1064 was slower than that of glucose, especially under 2% total sugar conditions (Fig. 4).

### Seeking the best candidate by combining engineering strategies

**Fig. 5:**
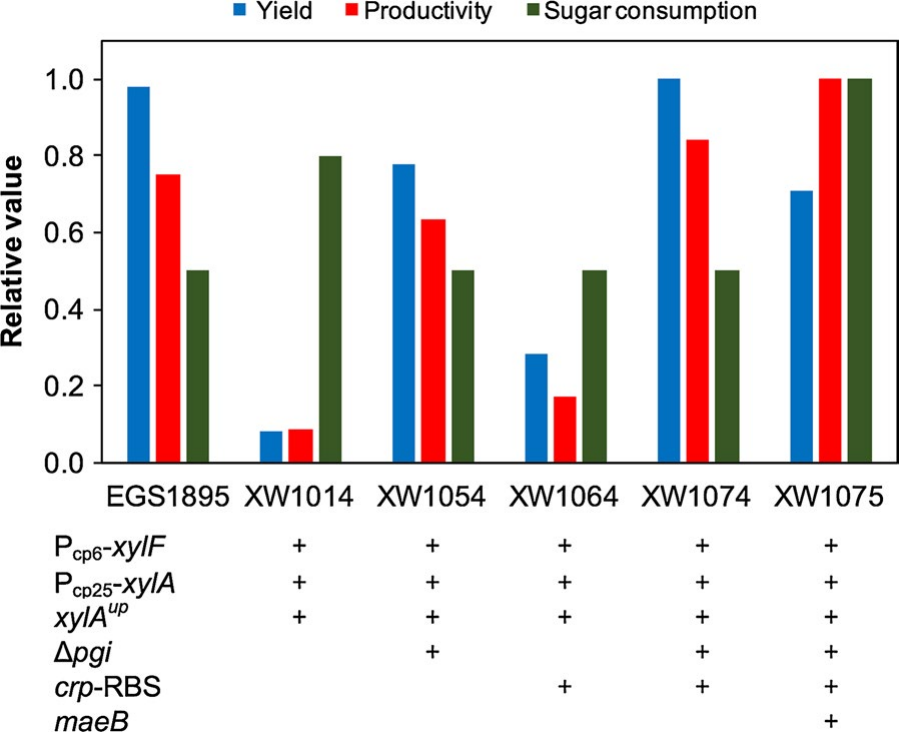
Summary comparison of methyl ketone production and sugar consumption for engineered strains. Methyl ketone yield, methyl ketone productivity, and sugar consumption period are each normalized to the maximum value among the six strains (for cultivation with 2% total sugars). Blue, methyl ketone yield from glucose + xylose consumed (g methyl ketones g^−1^ total sugars); red, methyl ketone productivity during the sugar consumption period (from onset of sugar consumption to > 90% total sugar consumption; g L^−1^ h^−1^); green, the reciprocal of sugar consumption period (as defined for productivity; the reciprocal was used to make the most favorable consumption phenotype approach 1 instead of 0 for ease of comparison)

Given the complementary features of the above strategies (Δ*pgi* and CRP downregulation) on cell growth and methyl ketone production, and the fact that they both effectively improved methyl ketone production in strain XW1014, we decided to combine these two strategies to obtain an additive effect. Overall, combining Δ*pgi* and CRP downregulation (strain XW1074; Table 1) created superior phenotypes in cell growth and methyl ketone production compared to use of either strategy alone (Fig. 4). This strain produced up to 570 mg L^−1^ methyl ketones at 1% total sugar conditions, but reached a higher titer at 2% total sugars (~ 1600 mg L^−1^) that was comparable to that of the control strain (EGS1895). Glucose and xylose were simultaneously consumed by strain XW1074 (Fig. 4) after a lag period, but utilization of xylose was still slower than that of glucose. Surprisingly, the added *maeB* overexpression (strain XW1075) dramatically improved sugar co-utilization (albeit with the same lag period, likely caused by *pgi* deletion; [34, 35]). As a result, strain XW1075 achieved synchronized consumption rates for glucose and xylose at both 1 and 2% total sugar conditions. Methyl ketone titers in strain XW1075 were up to 700 and 1100 mg L^−1^ at 1 and 2% total sugars, respectively. Thus, these two strains engineered with combined strategies (XW1074 and XW1075) represented a favorable phenotype displaying simultaneous utilization of glucose and xylose without substantially sacrificing methyl ketone production relative to the control strain (EGS1895) (Figs. 4, 5).

### Strain XW1075 performance during batch fermentation

**Fig. 6:**
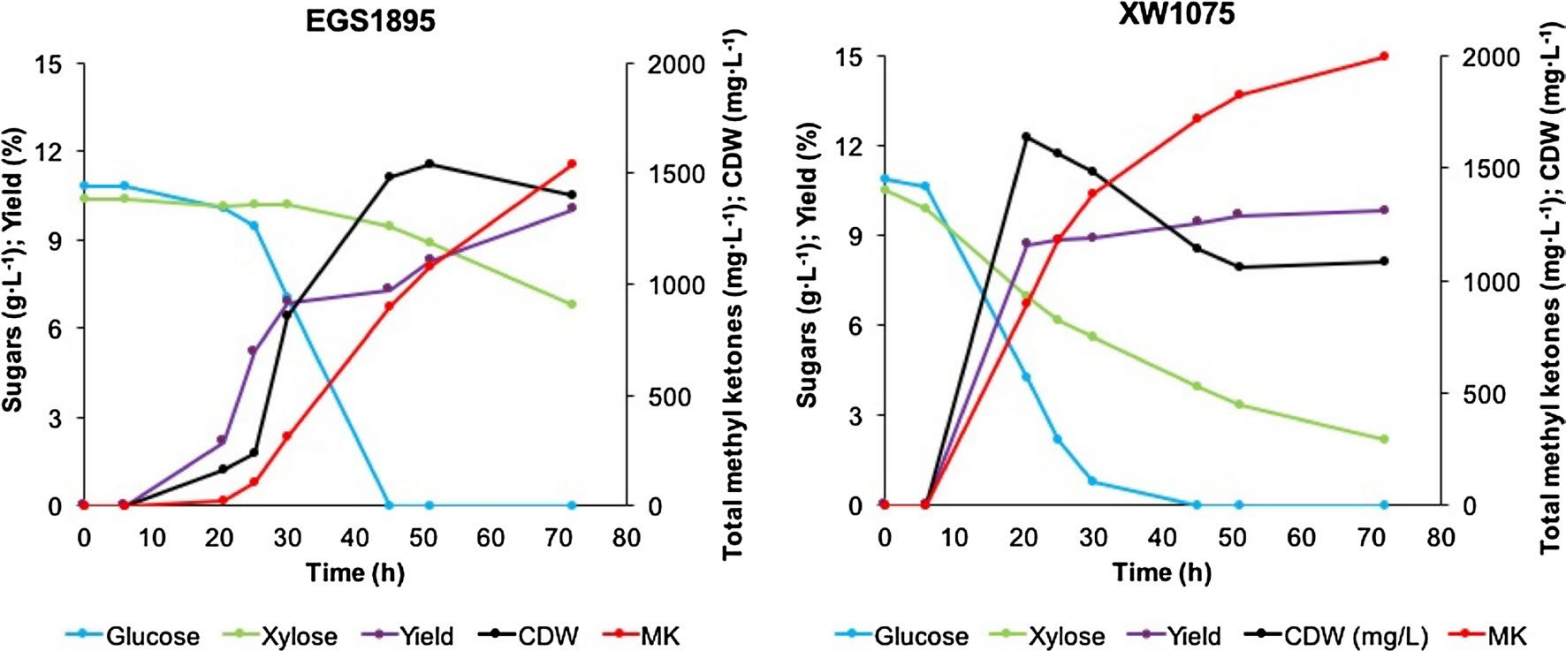
Batch fermentation of strains EGS1895 and XW1075 in 2-L bioreactors. Symbols: glucose, blue line; xylose, green line; cell dry weight (CDW), black line; methyl ketones, red line; yield, purple line

Strain XW1075 also compared favorably to control strain EGS1895 in batch fermentation mode. Glucose and xylose were utilized concurrently in strain XW1075 (albeit at unequal rates), whereas strain EGS1895 displayed a typical diauxic pattern, including sequential sugar utilization (Fig. 6). Correspondingly, strain XW1075 had a more consistent methyl ketone production yield (8.7–9.8%) than the control strain (6.9–10.0%). At 72 h, the methyl ketone titer of strain XW1075 was 2 g L^−1^, which was ca. 33% higher than that of strain EGS1895 (1.5 g L^−1^).

Comparison of the results in Fig. 6 with those of strains XW1075 and EGS1895 grown with pure glucose or xylose (Additional file 1: Figure S2) reveals that co-utilization of glucose and xylose in strain XW1075 enabled substantially better methyl ketone production than did utilization of either sugar alone. In fact, methyl ketone production was negligible for strain XW1075 utilizing either pure glucose or pure xylose (Additional file 1: Figure S2). Notably, strain EGS1895 also produced negligible methyl ketones when grown on pure xylose (Additional file 1: Figure S2), but produced substantial methyl ketones while metabolizing xylose after diauxic depletion of glucose (Fig. 6). From Figs. 4 and 6, it appears that glucose metabolism supported both growth and methyl ketone production in strain EGS1895, whereas xylose metabolism supported methyl ketone production but little or no growth.

## Conclusions

In this study, genetic manipulations were conducted to alleviate carbon catabolite repression in our most efficient methyl ketone-producing strain. A strain (XW1014) with constitutively expressed *xylA* and *xylF* plus a *xylA* promoter mutation showed well-synchronized glucose and xylose consumption rates. However, this newly acquired capability came at the expense of methyl ketone titer, which decreased fivefold. Further efforts were made to optimize methyl ketone production in this strain, and we found that chromosomal deletion of *pgi* (to enhance NADPH supply) and CRP downregulation by replacement of the native RBS both effectively improved methyl ketone production. Combining these strategies resulted in the most favorable overall phenotypes for simultaneous glucose–xylose consumption without compromising methyl ketone titer (Figs. 5, 6). Further optimization of performance will entail improved fermentation process conditions as well as additional genetic modifications.

## Additional file

**Additional file 1.** Supplementary figures and tables.

## Abbreviations

CCR: carbon catabolite repression
PEP: phosphoenolpyruvate
PTS: phosphotransferase
cAMP: cyclic AMP
CRP: cAMP receptor protein
PPP: pentose phosphate pathway
RBS: ribosomal binding site
TIR: translation initiation rate
MK: methyl ketones
CDW: cell dry weight
