# Supplementary material for: Engineering E. coli for simultaneous glucose–xylose utilization during methyl ketone production
Source: Microb Cell Fact. 2018 Jan 27;17:12. doi: 10.1186/s12934-018-0862-6 (PMC5787283; doi:10.1186/s12934-018-0862-6)
Supplement: Supplementary file 1 — Additional file 1. Supplementary figures and tables. [file 12934_2018_862_MOESM1_ESM.pdf]

ADDITIONAL FILE 1

Engineering *E. coli* for simultaneous glucose-xylose utilization during methyl ketone production

Xi Wang [1,2], Ee-Been Goh [1,2], Harry R. Beller* [1,2,3]

[1] Joint BioEnergy Institute (JBEI), 5885 Hollis St., Emeryville, CA 94608, USA

[2] Biological Systems & Engineering Division, Lawrence Berkeley National Laboratory, Berkeley, CA 94720, USA

[3] Earth & Environmental Sciences, Lawrence Berkeley National Laboratory, Berkeley, CA 94720, USA

Table S1 Primers used in this study

Target gene	Primer Name	Sequence (5'-3')
ptsG	ptsG_P1	GCGTGAGAACGTAAAAAAAGCACCCATACTCAGGAGCACTCTCAATTAT GATTCCGGGGATCCGTCGACC
	ptsG_P2	CTGCCTTAGTCTCCCCAACGTCTTACGGATTAGTGGTTACGGATGTACTC GTGTAGGCTGGAGCTGCTTCG
xylAF	xylAF_P1	TTTGAGCCTTCATAACGAAC
	xylAF_P2	CATGTAGTGAGGGGGCTGGTATAATCACATAGTACTTTTCACACAGGAA ACAGCTATGCAAGCCTATTTTGACCA
	xylAF_P3	ATCATCCGGAAATATCTGTGTCAAGAATAAACTCCCACATGATTCCGGGG ATCCGTCGACC
	xylAF_P4	ACCAGCCCCCTCACTACATGTCAAGAATAAACTGCCAAAGGTGTAGGCT GGAGCTGCTTC
	xylAF_P5	CACAGATATTTCCGGATGATATAATAACTGAGTACTGTTCACACAGGAAA CAGCTATGAAAATAAAGAACATTCTACTCACCC
	xylAF_P6	AACGTTGGTAAGCAGGAGTG
araE	araE_inact_F	ACGCGGCGTATGAATATGTTTGTTTCGGTAGCTGCTGCGGTCGCAGGATT ATTCCGGGGATCCGTCGACC
	araE_inact_R	GCCAGCACGATGCCGAGTGTGACCATCAACTGGTACATACTGATCATCTT TGTAGGCTGGAGCTGCTTCG
araB	araB_P1	CAAACCCTATGCTACTCCGT
	araB_P2	TTGGTAACGAATCAGACAATTGACGGCT
	araB_P3	CGTCAATTGTCTGATTCGTTACCAAGTGTAGGCTGGAGCTGCTTC
	araB_P4	ACATGTCAAGAATAAACTGCCAAAGATTCCGGGGATCCGTCGACC
	araB_P5	CTTTGGCAGTTTATTCTTGACATGTAGTGAGGGGGCT
	araB_P6	AAAGCTCGCACAGAATCACT
araF	araF_P1	AGGTAATGCGGCCTATTGACT
	araF_P2	TACCCATGCGGGATGTCTTCTT
	araF_P3	AAAAAGAAGACATCCCGCATGGGTAGTGTAGGCTGGAGCTGCTTC
	araF_P4	TGTGTCAAGAATAAACTCCCACATGATTCCGGGGATCCGTCGACC
	araF_P5	CATGTGGGAGTTTATTCTTG
	araF_P6	GACATAACGGCTGCCAGACCAAT
pgi	pgi_FRT1	TACAATCTTCCAAAGTCACAATTCTCAAAATCAGAAGAGTATTGCTAATG ATTCCGGGGATCCGTCGACC
	pgi_FRT2	GCCTTATCCGGCCTACATATCGACGATGATTAACCGCGCCACGCTTTATA TGTAGGCTGGAGCTGCTTCG
crp	crp_P1	TTCCCAGGTAGCGGGAAGCAT
	crp_P2	TGCAGGTCGACGGATCCCCGGAATCCAGATAACGCCGCTGTCTCT
	crp_P3	ATTCCGGGGATCCGTCGACC
	crp_P4	TGTAGGCTGGAGCTGCTTCG
	crp_P5	AACTTCGAAGCAGCTCCAGCCTACACTHTGGTGAAAGCTTATAACTGAG GMRAACCGCGTATGGTGCTTGGCAAACCGCA
	crp_P6	GTGGCAATGAGACAAGAACC

	crp_blk	AACTTCGAAGCAGCTCCAGCCTACACTCTGGAGAAAGCTTATAACAGAGG
pXW1677	1675_araC_P1	GGATTTTGGTCATGACTAGT
	1675_araC_P2	GACGTCGGAATTGCCAGCTG
	1675_araC_P3	CAGCTGGCAATTCCGACGTCACTTTTCATACTCCCGCCAT
	1675_araC_P4	AATTATTCACCTTCGTCGGCT
maeB	maeB_RBS_F	CAATCCGCTCGAGGAAGGAGATATACCATGGATGACCAGTTAAAACAAAGTGCAC
	maeB_R	GCCTGCAGGTCGACTTACAGCGGTTGGGTTTGCGCT

Table S2 Designed RBS sequences and associated Translation Initiation Rates (TIR) predicted by the RBS Library Calculator (Farasat et al., 2014)

No.	RBS Sequence	Predicted Translation Initiation Rate (au)
1	CT**C**TGGTGAAAGCTTATAACTGAGG**AG**AACCGCGT	7290
2	CT**C**TGGTGAAAGCTTATAACTGAGG**AA**AACCGCGT	2260
3	CT**T**TGGTGAAAGCTTATAACTGAGG**AG**AACCGCGT	807
4	CT**C**TGGTGAAAGCTTATAACTGAGG**CA**AACCGCGT	512
5	CT**C**TGGTGAAAGCTTATAACTGAGG**CG**AACCGCGT	312
6	CT**A**TGGTGAAAGCTTATAACTGAGG**AG**AACCGCGT	262
7	CT**T**TGGTGAAAGCTTATAACTGAGG**AA**AACCGCGT	250
8	CT**A**TGGTGAAAGCTTATAACTGAGG**AA**AACCGCGT	97
9	CT**T**TGGTGAAAGCTTATAACTGAGG**CA**AACCGCGT	52
10	CT**T**TGGTGAAAGCTTATAACTGAGG**CG**AACCGCGT	32
11	CT**A**TGGTGAAAGCTTATAACTGAGG**CA**AACCGCGT	13
12	CT**A**TGGTGAAAGCTTATAACTGAGG**CG**AACCGCGT	8

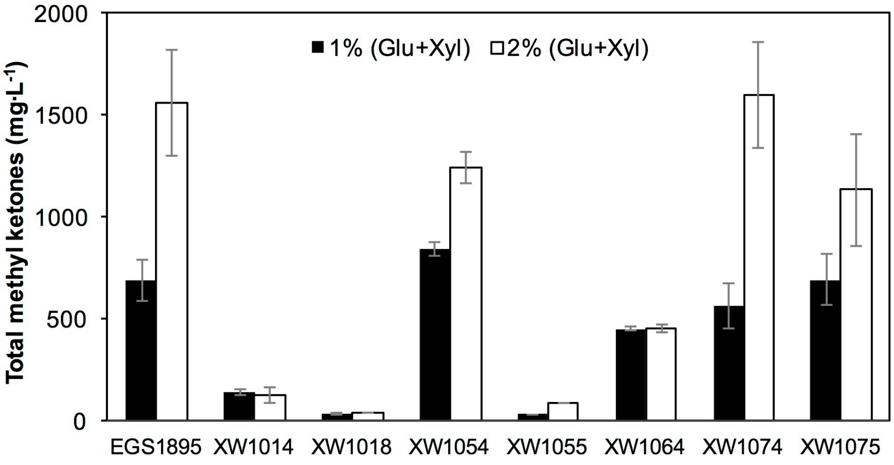

	EGS1895	XW1014	XW1018	XW1054	XW1055	XW1064	XW1074	XW1075
P_{cp6}-*xylF*		+	+	+	+	+	+	+
P_{cp25}-*xylA*		+	+	+	+	+	+	+
xylA^{up}		+	+	+	+	+	+	+
Δ*pgi*				+	+		+	+
crp-RBS						+	+	+
maeB			+		+			+

Fig. S1 Methyl ketone production titers by engineered strains at 1% and 2% sugar conditions. Error bars indicate one standard deviation.

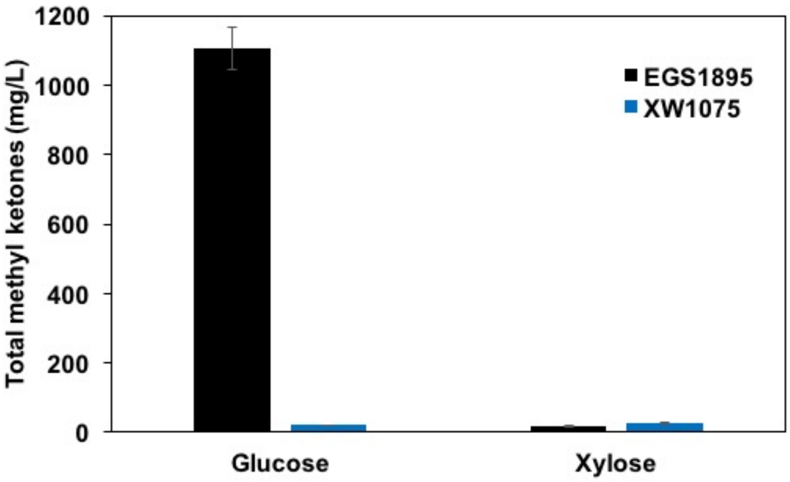

Fig. S2 Methyl ketone production titers for EGS1895 and XW1075 with 1% glucose or 1% xylose. Error bars indicate one standard deviation (*n* = 3).

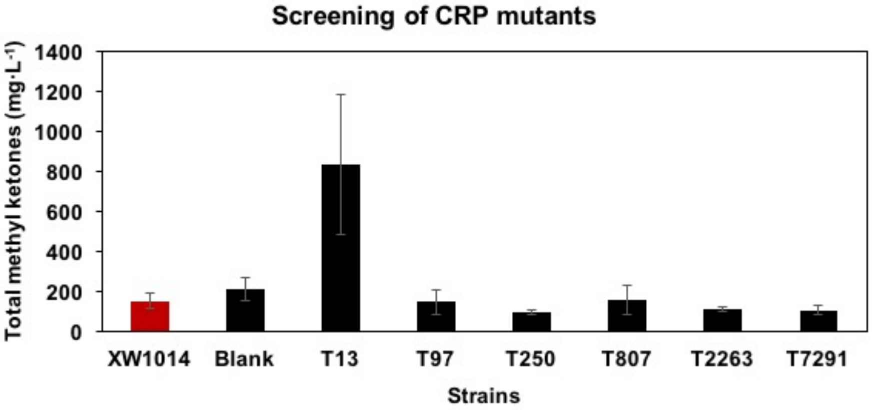

Fig. S3 Screening of CRP mutants by evaluating methyl ketone production. After transforming pEG1675, three biological replicates of each of the 7 mutants were picked for screening of methyl ketone production. The screening was conducted in 5 mL of M9-MOPS media in 50-mL test tubes after 96 h at 1% total sugar conditions. Strains with the λ-Red-generated 81-bp scar sequence but the native RBS of *crp* were used as the blank control. Error bars indicate one standard deviation (*n*=3) except for T13 (*n*=11).

References

Farasat, I., Kushwaha, M., Collens, J., Easterbrook, M., Guido, M., Salis, H. M., 2014. Efficient search, mapping, and optimization of multi-protein genetic systems in diverse bacteria. Mol. Syst. Biol. 10.
